# BAMBI and CHGA in Prion Diseases: Neuropathological Assessment and Potential Role as Disease Biomarkers

**DOI:** 10.3390/biom10050706

**Published:** 2020-05-02

**Authors:** Óscar López-Pérez, Marcos Bernal-Martín, Adelaida Hernaiz, Franc Llorens, Marina Betancor, Alicia Otero, Janne Markus Toivonen, Pilar Zaragoza, Inga Zerr, Juan José Badiola, Rosa Bolea, Inmaculada Martín-Burriel

**Affiliations:** 1 Laboratorio de Genética Bioquímica (LAGENBIO), Universidad de Zaragoza, Instituto Agroalimentario de Aragón-IA2, Instituto de Investigación Sanitaria Aragón-IISA, 50013 Zaragoza, Spain; oscarlzpz@gmail.com (Ó.L.-P.); marcosbermar@gmail.com (M.B.-M.); ahernaiz@unizar.es (A.H.); toivonen@unizar.es (J.M.T.); pilarzar@unizar.es (P.Z.); 2 Centro de Encefalopatías y Enfermedades Transmisibles Emergentes (CEETE), Universidad de Zaragoza, Instituto Agroalimentario de Aragón-IA2, Instituto de Investigación Sanitaria Aragón-IISA, 50013 Zaragoza, Spain; mbetancorcaro@gmail.com (M.B.); aliotgar@hotmail.com (A.O.); badiola@unizar.es (J.J.B.); rbolea@unizar.es (R.B.); 3 Centro de Investigación Biomédica en Red de Enfermedades Neurodegenerativas (CIBERNED), Institute Carlos III, 28029 Madrid, Spain; franc.llorens@gmail.com; 4 Instituto de Investigación Biomédica de Bellvitge (IDIBELL), L’Hospitalet de Llobregat, 08908 Barcelona, Spain; 5 Department of Neurology, Clinical Dementia Center and National Reference Center for CJD Surveillance, University Medical School, 37075 Göttingen, Germany; ingazerr@med.uni-goettingen.de; 6 Department of Biological Sciences, Centre for Prions and Protein Folding Diseases, University of Alberta, Edmonton, AB T6G 2R3, Canada

**Keywords:** prion disease, scrapie, Creutzfeldt-Jakob disease, sheep, transgenic mice, biomarkers, neuroinflammation, bone morphogenetic protein and activin membrane-bound inhibitor, chromogranin A

## Abstract

Prion diseases affect both animals and humans. Research in the natural animal model of the disease could help in the understanding of neuropathological mechanisms and in the development of biomarkers for human pathologies. For this purpose, we studied the expression of 10 genes involved in prion propagation in vitro in the central nervous system of scrapie-infected sheep. Dysregulated genes (*BAMBI* and *CHGA*) were further analysed in a transgenic murine model (Tg338) of scrapie, and their protein distribution was determined using immunohistochemistry and Western blot. Their potential as biomarkers was finally assessed using enzyme-linked immunosorbent assay (ELISA) in cerebrospinal fluid (CSF) of scrapie sheep and Creutzfeldt-Jakob disease (CJD) patients. Protein BAMBI was upregulated in highly affected brain areas and CHGA was overexpressed along the brain in both models. Moreover, BAMBI and CHGA immunostaining scores strongly correlated with spongiosis and microgliosis in mice. Finally, levels of BAMBI were significantly higher in the CSF of clinical sheep and CJD patients. In addition to their potential as biomarkers, our work confirms the role of BAMBI and CHGA in prion neuropathology in vivo, but besides prion replication, they seem to be involved in the characteristic neuroinflammatory response associated to prion infection.

## 1. Introduction

Transmissible spongiform encephalopathies (TSE), or prion diseases, are fatal neurodegenerative disorders caused by prions [1]. These pathologies affect humans and animals and share common pathological features such as accumulation of abnormal conformers (PrP^Sc^) of the cellular prion protein (PrP^c^) in the central nervous system (CNS), which leads to neuronal dysfunction and cell death. Ovine scrapie was the first TSE described and constitutes one of the most widely studied models of these pathologies [2]. 

Transcriptomic studies have revealed differentially expressed genes that could be involved in the pathogenesis of prion diseases and are potential targets for therapies in humans [3,4,5]. Most of these studies were done using mice infected with different prion strains [4]. Our group developed several transcriptomic analyses in the CNS of scrapie-infected sheep that confirmed changes described in murine scrapie, Creutzfeldt-Jakob disease (CJD) and in other neurodegenerative diseases like Alzheimer’s disease (AD); those changes were associated with neuropathological alterations. Similarities observed between ovine scrapie and human neurodegenerative diseases support research in the natural animal model of the disease for the development of biomarkers for diagnosis and therapy and for the study of the molecular mechanisms of prion neuropathology [6,7,8]. 

In vitro models are also useful for the identification of biomarkers or genes involved in TSE neuropathology. A transcriptomic comparison between prion-resistant revertants and highly susceptible clones of the mouse neuroblastoma N2a cells revealed a genomic signature associated with prion replication and susceptibility [9]. The described regulatory network includes genes encoding proteins with a known role in extracellular matrix (ECM) remodeling, suggesting that genes involved in ECM homeostasis affect prion conversion in vitro. Similarly, a genomic study performed in natural scrapie-infected sheep at the early stage of the disease revealed a downregulation of genes implicated in the organization of the ECM [7]. Some of these genes (*FN1* and *MMP14*) are also expressed differently in permissive and poorly permissive ovine microglial cells [10]. 

The aim of the present study was to evaluate whether the genes described as potentially involved in prion protein replication in vitro could also play a role in the neuropathology of these diseases in vivo. For this purpose, we selected a battery of ten of those genes that were downregulated in N2a clones permissive to prion replication [9] and quantified their expression in the medulla oblongata of sheep naturally infected with classical scrapie. The expression and distribution of proteins encoded by differentially expressed genes, i.e., *BAMBI* (BMP (bone morphogenetic protein) and activin membrane-bound inhibitor) and *CHGA* (chromogranin A), were subsequently evaluated in the CNS of scrapie-affected sheep and transgenic mice. Chromogranin A is an acidic protein expressed by cells of the endocrine and neuroendocrine systems. Its physiological role is related with granule biogenesis, calcium homeostasis and may play an autocrine role at the CNS [11]. This protein has been used as a biomarker of several diseases, including neurodegenerative diseases [11,12]. Chromogranin B but not A has been associated with prion deposits in CJD [13]. On the other hand, BAMBI is a transmembrane pseudoreceptor that negatively regulates TGF-β (transforming growth factor-beta) signalling by preventing the formation of active receptor complexes [14,15]. In adult mice, *Bambi* transcripts are expressed in several regions of the CNS involved in pain processing [16]. In this work, we investigated the relationship between these proteins and the appearance of the main prion-related lesions and their potentiality as prion disease biomarkers.

## 2. Materials and Methods 

### 2.1. Animals and Samples

For each analysis performed, different control and naturally scrapie-infected sheep groups were sampled or used from previous studies (Ethical code: PI38/15 and PI40/15) [17,18]. All animals were female Rasa Aragonesa sheep displaying the ARQ/ARQ genotype for the *PRNP* gene. The detailed characteristics of these groups are shown in Table S1 and Table S2.

A transgenic mouse model (Tg338) overexpressing the highly susceptible VRQ (valine (V) at codon 136, arginine (R) at codon 154, and glutamine (Q) at codon 171) allelic variant of the ovine *PRNP* gene [19] was used to evaluate the gene expression and protein distribution of *Bambi* and *Chga* in the CNS. The experimental mice were intracerebrally inoculated into the right frontal lobe with Tg338-adapted prions derived from classical scrapie sheep material and were euthanized at preclinical (*n* = 6) or clinical (*n* = 6) stages of the disease. Two other groups of mock-inoculated Tg338 mice (*n* = 6 each) were sacrificed at the same time points and used as age-matched controls. The experimental groups and sample collection are more extensively described in a previous work [20].

The intensity of the BAMBI signal was also quantified in human cerebrospinal fluid (CSF) samples (Table S2). This study included 58 patients recruited at Clinical Dementia Center Gottingen and at the National Reference Center for CJD Surveillance at the Department of Neurology of the University Medical Center of Göttingen, Germany. Patients diagnosed with probable or definite sporadic CJD according to established diagnostic criteria were considered for inclusion in the study (*n* = 34). The neurological disease control group (ND) (*n* = 24) was composed of patients with either clinically or pathologically defined neurological disease with non-neurodegenerative etiology (psychiatric disorders, epilepsy, autoimmune diseases, encephalitis, alcohol abuse disorder, headache and alternative neurologic conditions). Lumbar punctures were performed at the time of the first routine diagnostic work up.

The care and use of experimental animals were performed in strict accordance with the national law (R.D. 53/2013), and all experimental procedures were approved by the Ethics Committee for Animal Experiments of the University of Zaragoza (Permit Number: PI38/15 and PI40/15). The study of human CSF cases was conducted according to the revised Declaration of Helsinki and Good Clinical Practice guidelines and with informed written consent provided by all patients or by their next of kin in the case of cognitive impairment. All procedures in human cases were approved by the Ethical Committee of the University of Gottingen (Ref: 11/11/93).

### 2.2. Gene Expression Analysis

Ten genes (*BAMBI, CHGA, DLC1, FN1, GALT, IL11RA, ITGA8, LRRN4, PAPSS2* and *RGS4*), described to be involved in prion replication in vitro [9] were selected for the analysis of their expression profile in the medulla oblongata (Mo) of sheep, one of the most affected brain areas in classical scrapie [17,21]. In addition, gene expression of murine *Bambi* and *Chga* was quantified in mesencephalon (Mes) of Tg338 mice. We used this tissue because it shows the most abundant accumulation of PrP^Sc^ and the highest scores of spongiform changes in this mouse model [20].

Total RNA was obtained from 100 mg of ovine Mo preserved in RNAlater using a RNeasy Lipid Tissue Mini kit (QIAGEN^®^, Venlo, Netherlands) and following the manufacturer’s recommended protocol. Genomic DNA was digested using a Turbo DNA-free kit (Ambion, Waltham, Massachusetts, USA). Complementary DNA (cDNA) was obtained from 500 ng of total RNA using a SuperScript First-Strand Synthesis System kit (Invitrogen, Waltham, Massachusetts, USA). Final cDNA was diluted 1:10 in water for further analyses. In Tg338 mice, total RNA was isolated from RNAlater-preserved Mes using a Direct-Zol^TM^ RNA kit (Zymo Research, Irvine, California, USA). Retrotranscription was performed from 200 ng of total RNA using qScript^TM^ cDNA Supermix (Quanta Biosciences^TM^, Beverly, Massachusetts, USA). Resulting cDNA was diluted 1:5 in water.

The quantitative polymerase chain reaction (qPCR) primers for the amplification of the ovine genes were designed using Primer Express 2.0 software (Applied Biosystems, Waltham, Massachusetts, USA). Table S3 shows the sequences of these primers. PCR reactions were performed using SYBR^®^ Green (Thermo Fisher Scientific, Waltham, Massachusetts, USA), and PCR was carried out in a StepOne Real-Time PCR System (Thermo Fisher Scientific) using universal conditions. All reactions were run in triplicate in a total volume of 10 µL, using 2 µL of diluted cDNA and 300 nM of each primer. The expression of three housekeeping genes (*G6PDH, GAPDH*, and *HPRT*) was used to normalize results. The selected housekeeping genes are stable in scrapie-affected sheep brain [22]. Raw fluorescence data obtained for each PCR reaction were analysed with LinRegPCR software [23], which used the mean PCR efficiency per amplicon and the quantification cycle (Cq) value per sample to calculate a starting concentration per sample, expressed in arbitrary fluorescence units. Relative gene expression quantification was subsequently determined using normalized data after log-transformation.

Gene expression of *Bambi* and *Chga* in Tg338 mice was quantified using qPCR in Mes using the Mm03024088_g1 and Mm00514341_m1 assays (Thermo Fisher Scientific), respectively, in a StepOne Plus (Applied Biosystems). Expression levels were normalized with *Sdha* and *H6pd* housekeeping genes, which were amplified using the Mm01352366_m1 and Mm00557617_m1 assays (Thermo Fisher Scientific), respectively. Relative quantification was performed using the 2^-ΔΔCt^ method.

Differences between experimental groups were evaluated using an unpaired Student’s *t*-test (two tailed). In addition, the expression profile of the ten genes in ovine tissues were compared between control and scrapie sheep using a two-way analysis of variance (ANOVA) followed by a Bonferroni post-test. Pearson r and Spearman ρ correlations were calculated to detect any relationship between relative expression values and animal age (days) and to identify associations between gene expression levels and histopathological lesions in Mo.

### 2.3. Immunohistochemical Determination of BAMBI and CHGA

Proteins encoded by differentially expressed genes (*BAMBI* and *CHGA*) were selected for to analyse their expression and distribution in the CNS of scrapie-infected sheep using immunohistochemistry (IHC). Immunostaining of BAMBI and CHGA was determined in four formalin-fixed paraffin-embedded CNS sections: frontal cortex (Fc), thalamus (T), cerebellum (Cbl) and Mo. Heat pre-treatment (96 °C for 20 min) in EnVision Flex target retrieval solution at low and high pH was used in a PTLink PT100 (Dako, Santa Clara, California, CA, USA) for BAMBI and CHGA, respectively. Rabbit polyclonal antibodies were used to detect BAMBI (1:1000 dilution at room temperature (RT) for one hour (h); PA5-38027; ThermoFisher, Waltham, MA, USA) and CHGA (1:200 dilution at RT for 1 h; PA5-16685; ThermoFisher). The reaction specificity was ensured by including a background control section without primary antibody in each staining procedure. EnVision Polymer anti-rabbit (Dako) was used to detect primary antibodies and 3.3′-diaminobenzidine as chromogen (Dako).

In Tg338 mice, immunostaining of BAMBI and CHGA was evaluated in nine CNS areas [24]: Fc, septal area (Sa), thalamic cortex (Tc), hippocampus (Hc), T, hypothalamus (Ht), Mes, Cbl (which includes molecular layer (Ml), Purkinje layer (Pl), granular layer (Gl), white matter (Wm) and deep cerebellar nuclei (DCN)) and Mo. The IHC protocol used was similar to that described for the ovine brain samples, but in this case, BAMBI immunolabelling was performed at 4 °C overnight. 

In both cases, IHC was performed using scrapie and control tissues in the same assay to guaranty quantification reliability. Stained sections were examined with a ZEISS Axioskop 40 optical microscope and scored semi-quantitatively from 0 (no immunostaining) to 5 (strongly stained areas) as previously described [25,26,27]. Scoring for immunolabelling was performed blind by two different pathologists making subjective evaluation. The same two pathologists evaluated each brain region using low magnification (10×) and moving through the entire tissue to obtain comparable data from the proteins studied. In sheep, the layers or nuclei of each brain region were analysed separately, and the final score was calculated as the mean of those layers or nuclei. In Tg338 mice, each area was investigated globally as a region for the scoring, except for Cbl, whose layers were analysed separately. A higher magnification (40×) was used to describe and identify the location of staining. The reliability of this subjective quantification was confirmed using Western blot in a previous work [17]. Immunohistochemical differences between the experimental groups were assessed using the non-parametric Mann-Whitney U test. Robustness of differences observed was verified comparing IHC profiles using two-way ANOVA and post-test Bonferroni correction.

### 2.4. Western Blot Analysis

The expression of BAMBI and CHGA was quantified in brain lysates from scrapie and control sheep using Western blot. Briefly, 25 µg of total protein obtained from CNS tissues (Fc, T, Cbl and Mo) were subjected to 10% sodium dodecyl sulphate-polyacrylamide gel electrophoresis (SDS-PAGE) and transferred to polyvinylidene fluoride (PVDF) membranes (GE Healthcare, Chicago, Illinois, USA). After blocking at RT for 1 h, the membranes were incubated at 4 °C overnight using the primary antibodies described above for IHC (both 1:1000 dilution). In addition, mouse monoclonal β-actin (1:200 dilution; C4, sc-47778; Santa Cruz Biotechnology, Dallas, Texas, USA) was used to normalize results. Next, the membranes were incubated for 1 h at RT with a HRP-conjugated secondary antibody (1:4000 dilution, goat anti-rabbit IgG-HRP for BAMBI and CHGA, or goat anti-mouse for β-actin; Santa Cruz Biotechnology). Western blots were developed using the ECL Plus Western Blotting system (GE Healthcare) and visualized with the VersaDoc imaging system (Bio-Rad, Hercules, California, CA, USA).

The quantification of Western blot results was performed using an ImageJ 1.50 image-analysis software package (Psion Image, NIH) [28]. Beta-actin density was used as normalizer. Differences between experimental groups in Western blot were evaluated using the Student’s *t*-test.

### 2.5. Histopathological Study of Central Nervous System Tissues

Scrapie-related histopathological lesions (neuropil spongiosis, intraneuronal vacuolation and PrP^Sc^ deposition) were evaluated in the aforementioned brain areas of sheep and Tg338 mice in previous works [17,20]. Published scores were used to establish a relationship between the immunohistochemical distribution of BAMBI and CHGA and prion pathology. In addition, microgliosis was evaluated here using IHC, using a primary antibody goat polyclonal Iba1 (1:600 dilution at 4 °C overnight; ab5076; Abcam) and a biotinylated multilink swine anti-goat/mouse/rabbit immunoglobulin G (1:200 dilution at RT for 1 h; Dako) as a secondary antibody. A Vectastain ABC kit (Vector Labs Inc., Chicago, Illinois, IL, USA) was used according to the manufacturer´s instructions to visualize the binding of the primary antibody. Correlations between scrapie-related lesions and BAMBI and CHGA immunostaining were evaluated using the non-parametric Spearman ρ correlation.

### 2.6. Quantification of BAMBI and CHGA in Cerebrospinal Fluid 

The levels of BAMBI and CHGA in CSF of scrapie and control sheep were determined using quantitative ELISA kits (MBS7226525 and MBS033202; MyBioSource, San Diego, California, CA, USA) according to the manufacturer´s recommendations. Briefly, 50 µL of the samples were incubated in duplicate in pre-coated plates with 50 µL of HRP-conjugate reagent for 1 h at 37 °C and 100 µL of a substrate for HRP enzyme for 15 min at 37 °C. Subsequently, 50 µL of a stop solution was added to end the enzyme-substrate reaction and the intensity of the colour in the wells was measured spectrophotometrically at 450 nm using a SPECTROstar Nano microplate reader (BMG Labtech, Ortenberg, Germany). Inter- and intra-coefficient of variation were 6.4% and 4.9%, respectively, for BAMBI. Only one ELISA reaction was performed for CHGA, its intra-coefficient of variation being 5%.

Similarly, BAMBI was quantified in human CSF of ND and CJD patients using an ELISA kit from Sino Biological (Beijing, China) following the manufacturer’s instructions. Inter- and intra-coefficient of variation were 10% and 6% respectively. In addition, we determined total-tau (t-tau) concentrations and 14-3-3 positivity in the CSF to verify that the CJD cases present a typical CSF prion biomarkers profile. t-tau was quantified using an ELISA kit from Fujirebio-Europe (Ghent, Belgium) and the presence of 14-3-3 protein was analysed using Western blot as described previously [29]. 

According to distributional features, the Student´s *t*-test was used to determine statistical differences between control and scrapie sheep and between ND and CJD cases in ELISA analyses. Pearson´s r correlation was used to assess associations between continuous biomarker levels. We used IBM^®^ SPSS^®^ statistics 22 software for all data analysis and GraphPad Prism version 6.0 to perform the graphs. In all graphs, data are expressed as means ± standard deviation. The results were considered significant at *p* < 0.05.

## 3. Results

### 3.1. Dysregulation of BAMBI and CHGA in the Central Nervous System of Scrapie Animals

**Figure 1:**
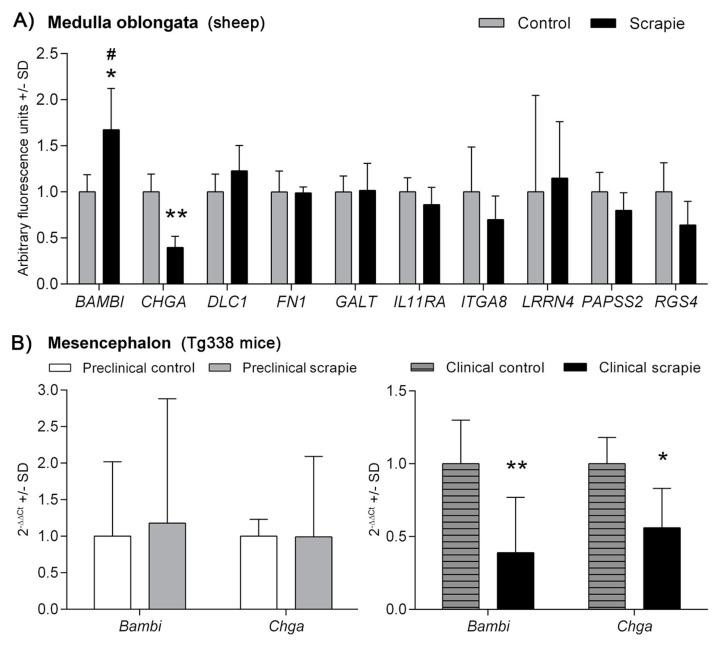
mRNA expression profiles of prion replication-related genes in (**A**) the medulla oblongata of sheep naturally-infected with scrapie (black bars) and control animals (grey bars) and in (**B**) mesencephalon of preclinical (grey bars) and clinical (black bars) scrapie-infected Tg338 mice. White bars and grey-striped bars display expression levels in controls for preclinical and clinical mice, respectively. Relative expression levels are expressed as means ± standard deviation. Relative expression values (*y*-axis) are expressed as arbitrary units. Results were normalized using the geometric mean of the expression of *G6PDH*, *GAPDH* and *HPRT* housekeeping genes in sheep and the expression of *Sdha* and *H6pd* in Tg338 mice. Differences between groups were evaluated with the Student´s *t*-test (* *p* < 0.05 and ** *p* < 0.01). Significant differences after Bonferroni correction for multiple tests are shown as # *p* < 0.05.

Figure 1A shows the relative gene expression values of the 10 genes analysed in Mo of scrapie and control sheep. Whilst a trend to downregulation was observed in half of the analysed genes, only *CHGA* displayed a statistically significant negative regulation in these tissues (*p* < 0.01). On the contrary, *BAMBI* was upregulated in scrapie medullae (*p* < 0.05). Two-way ANOVA revealed a significant effect of the disease in the expression profile of the ten genes, which explained 17% of variation (*p* = 0.02), and the Bonferroni post-test confirmed the effect of scrapie in the upregulation of *BAMBI* (*p* < 0.05). Due to the difficulty of finding controls with the same genotype, animals used as controls were obtained from culling sheep from a scrapie-free flock, which were of the same sex and breed, but significantly older than the scrapie-affected animals (Table S1). The only gene that displayed a significant correlation between its expression level and age, in the total set of animals, was *CHGA* (Pearson r = 0.827, *p* = 0.006). However, this correlation was lost within individual groups (r = 0.68, *p* = 0.32 in controls and r = 0.03, *p* = 0.99 in scrapie sheep). 

Scrapie pathology was detected consistently in Mo of scrapie sheep but not in control animals (Figure S1). Using the total set of animals, we only detected significant correlations between histopathological lesions and the expression of those genes that displayed significant expression changes between groups (Table S4). Once controls were removed from the correlation study, *BAMBI* transcript levels positively correlated with PrP^Sc^ deposition in scrapie animals (r = 0.98, *p* = 0.003) and spongiosis scores showed significant positive correlation with the expression levels of *GALT* (r = 0.962, *p* = 0.009), *PAPSS2* (r = 0.968, *p* = 0.007) and *RGS4* (r = 0.938, *p* = 0.018).

Transcripts from *Bambi* and *Chga* genes were also quantified in Mes of clinical and preclinical Tg338 mice and their control groups (Figure 1B). A significant downregulation was observed for both *Bambi* (*p* < 0.01) and *Chga* (*p* < 0.05) in the clinical scrapie mice compared to their selected age-matched control group. Significant changes were not detected at the preclinical stage of the disease. 

### 3.2. Overexpression of BAMBI and CHGA Proteins in the Central Nervous System of Scrapie Animals

**Figure 2:**
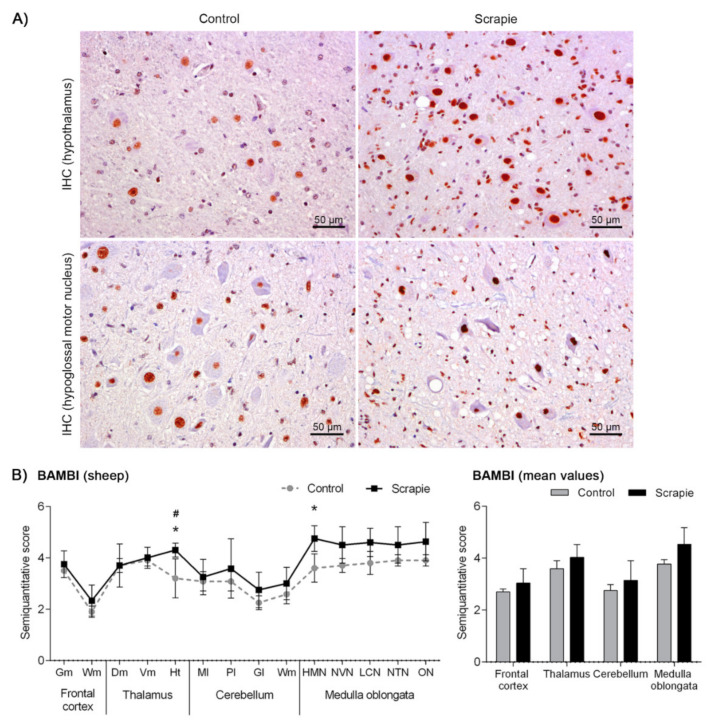
Immunostaining patterns and semi-quantitative scoring of BAMBI in the central nervous system of scrapie-infected sheep. (**A**) Representative images of BAMBI immunostaining in the hypothalamus and hypoglossal motor nucleus (50 µm). This protein displayed a homogeneous intranuclear pattern in both neurons and glial cells in scrapie and control sheep, although the amount of immunopositive cells and the intensity of immunolabelling were moderately larger in some regions of the clinical sheep. (**B**) Comparative graphs between scrapie (black bars) and control sheep (grey bars). Score values (from 0: negative, to 5: staining present at its maximum intensity) evaluated in the frontal cortex, thalamus, cerebellum and medulla oblongata. Abbreviations: grey matter (Gm), white matter (Wm), dorsomedial (Dm), ventromedial (Vm), hypothalamus (Ht), molecular layer (Ml), Purkinje layer (Pl), granular layer (Gl), hypoglossal motor nucleus (HMN), dorsal nucleus of the vagus nerve (NVN), lateral cuneate nucleus (LCN), nucleus of the trigeminal nerve spinal tract (NTN), olivary nucleus (ON). Significant differences were determined using the Mann Whitney U test (*p < 0.05). Significant differences after Bonferroni correction for multiple tests are shown as # *p* < 0.05.

Immunostaining of BAMBI displayed a homogeneous intranuclear pattern in both neurons and glial cells in all brain areas of scrapie and control sheep (Figure 2A). However, the amount of immunopositive cells and the intensity of immunolabelling were moderately higher in some CNS regions of scrapie-affected sheep (Figure 2B), this increment being statistically significant in Ht (*p* = 0.047) and the hypoglossal motor nucleus (HMN) in Mo (*p* = 0.031). Two-way ANOVA comparing IHC patterns in control and scrapie tissues confirmed the effect of the disease in the expression of this protein, which explained 53.05% of variation (*p* < 0.0001), whereas 8.77% corresponded to the effect of the area analysed (*p* < 0.0001). Bonferroni post-test correction confirmed the significant difference of BAMBI immunostaining in Ht (*p* < 0.05).

**Figure 3:**
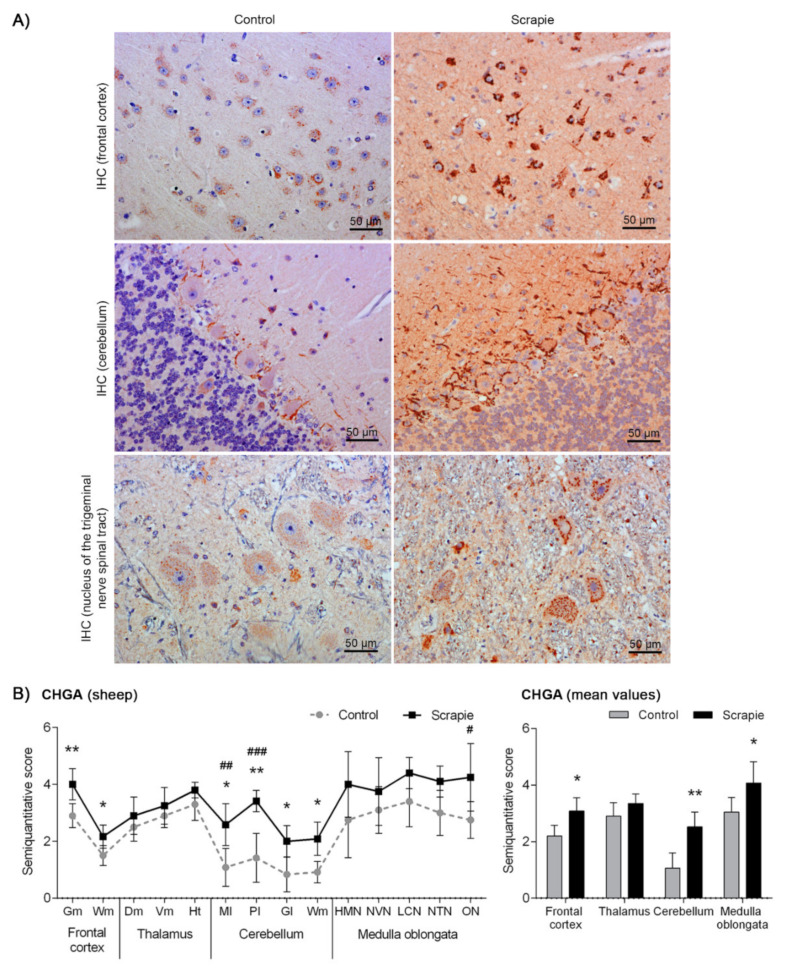
Immunostaining patterns and semi-quantitative scoring of CHGA in the central nervous system of scrapie-infected sheep. (**A**) Representative images of CHGA immunostaining in the frontal cortex, cerebellum and nucleus of the trigeminal nerve spinal tract (50 µm). This protein was found forming coarse intracytoplasmic aggregates in the frontal cortex, occupying almost the entire cytoplasm, whereas it was observed as fine particulate deposits in the medulla oblongata. These granular-like deposits were more prominent and intense in the scrapie sheep group. In the cerebellum, CHGA displayed a synaptic-like and a perineuronal arrangement in the Purkinje layer, being remarkably stronger in the scrapie-affected animals. (**B**) Comparative graphs between scrapie (black bars) and control sheep (grey bars). Score values (from 0: negative, to 5: staining present at its maximum intensity) evaluated in the frontal cortex, thalamus, cerebellum and medulla oblongata. Abbreviations: grey matter (Gm), white matter (Wm), dorsomedial (Dm), ventromedial (Vm), hypothalamus (Ht), molecular layer (Ml), Purkinje layer (Pl), granular layer (Gl), hypoglossal motor nucleus (HMN), dorsal nucleus of the vagus nerve (NVN), lateral cuneate nucleus (LCN), nucleus of the trigeminal nerve spinal tract (NTN), olivary nucleus (ON). Significant differences were determined using the Mann Whitney U test (* *p* < 0.05 and ** *p* < 0.01). Significant differences after Bonferroni correction for multiple tests are shown as #*p* < 0.05, ## *p* < 0.01 and ### *p* < 0.001.

Immunostaining of CHGA displayed different immunohistochemical patterns in sheep depending on the brain area analysed (Figure 3A). Primarily, this protein formed intracytoplasmic aggregates. In Fc and T, these aggregates were coarse, occupying almost the entire cytoplasm, whereas they displayed fine particulate deposits in Mo. These granular-like deposits were more prominent and intense in the scrapie sheep group. In addition, a linear punctiform immunostaining was observed in the neuropil throughout the brain. In Cbl, we observed a synaptic-like and a perineuronal arrangement of this protein in the Purkinje cells, being remarkably stronger in the scrapie-affected animals (*p* = 0.006). In addition, these cells showed a homogeneous intracytoplasmic staining, which was minimal or absent in the control group and more intense in the scrapie-infected sheep. Similar to BAMBI, the staining of CHGA was stronger in scrapie brains (Figure 3B), being significantly higher in Fc (*p* = 0.015), Cbl (*p* = 0.004) and Mo (*p* = 0.031). In this case, disease status explained 50.81% of variation and tissues 17.96% (two-way ANOVA test, *p* < 0.0001 in both cases). Bonferroni post-test confirmed significant differences between control and scrapie tissues in Cbl (molecular layer, *p* < 0.01; Purkinje layer, *p* < 0.001) and Mo (olivary nucleus, *p* < 0.05).

**Figure 4:**
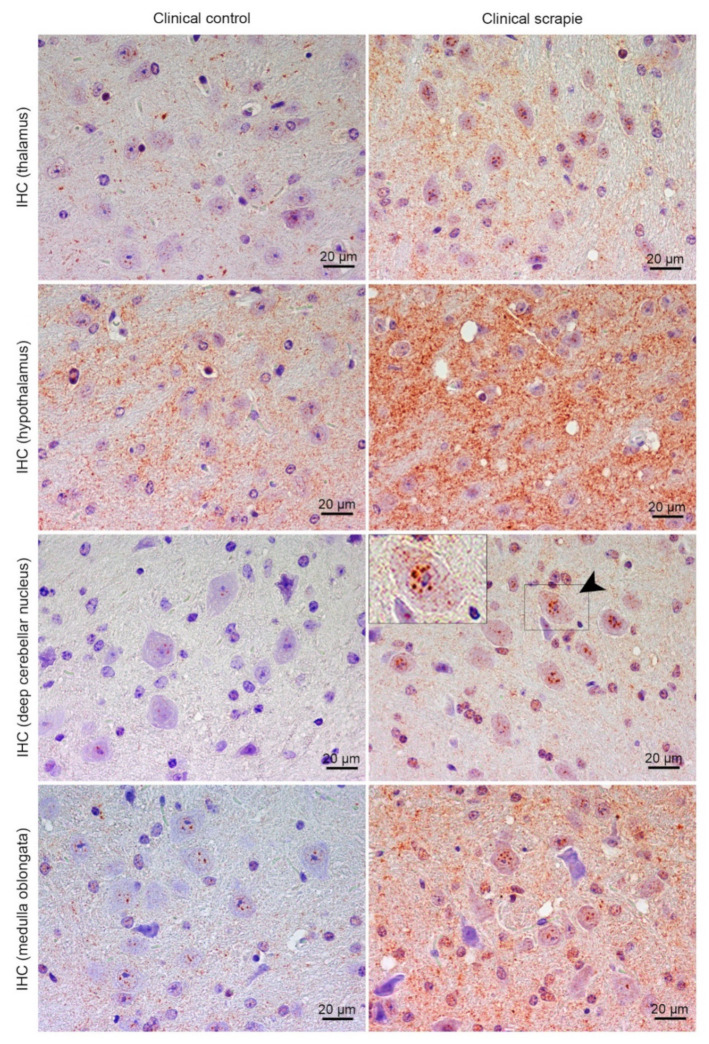
Immunostaining patterns of BAMBI in the central nervous system of scrapie-infected Tg338 mice. Figure shows representative images of BAMBI immunostaining in the thalamus, hypothalamus, deep cerebellar nucleus and medulla oblongata (20 µm). Deposition type of BAMBI consisted of coarse granules found in the nuclei of cells, which were more evident in neurons of the thalamus, deep cerebellar nuclei of cerebellum (arrowhead and detail) and medulla oblongata. In addition, a diffuse neuropil staining was observed, remarkably strong in the hypothalamus but also present in the other brain regions studied.

**Figure 5:**
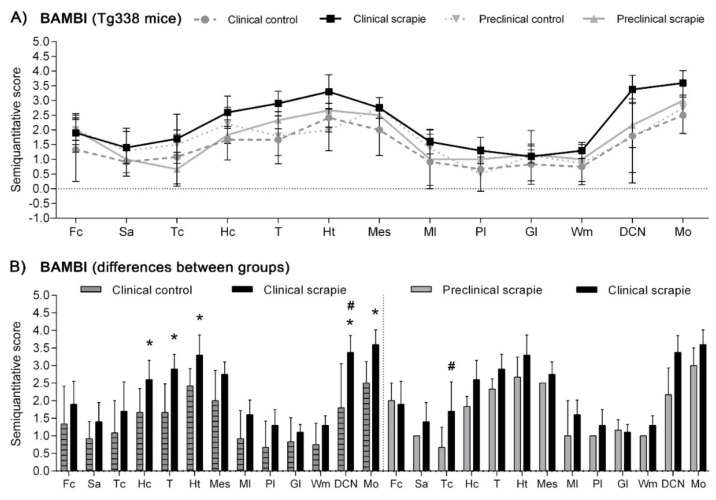
Semi-quantitative scoring of BAMBI in the central nervous system of scrapie-infected Tg338 mice. Figure includes a global graph (**A**) and comparative graphs (**B**) between clinical group (black bars) with preclinical (grey bars) and control group (grey-striped bars). Comparative graphs between the preclinical group and their control group, and between control groups, are not shown because no significant differences were detected. Score values (from 0: negative, to 5: staining present at its maximum intensity) evaluated in the frontal cortex (Fc), septal area (Sa), thalamic cortex (Tc), hippocampus (Hc), thalamus (T), hypothalamus (Ht), mesencephalon (Mes), cerebellum (which includes molecular layer (Ml), Purkinje layer (Pl), granular layer (Gl), white matter (Wm) and deep cerebellar nuclei (DCN)) and medulla oblongata (Mo). The differences between the experimental groups were determined using the Mann Whitney U test (* *p* < 0.05). Significant differences after Bonferroni correction for multiple tests are shown as # *p* < 0.05.

In Tg338 mice, BAMBI deposition type consisted of coarse granules found in the nuclei of cells (Figure 4). This granular pattern was most evident in T, DCN and Mo. In addition, a diffuse neuropil staining was observed, remarkably strong in Ht but also present in the other brain regions studied. Compared to controls, clinical mice showed higher accumulation of BAMBI in almost all brain structures (Figure 5A). In particular, this increment was statistically significant in Hc (*p* = 0.034), T (*p* = 0.026), Ht (*p* = 0.039), DCN (*p* = 0.031) and Mo (*p* = 0.023) of clinically-infected mice compared to their control group (Figure 5B). No significant differences were detected in preclinical animals. However, the effect on the disease was lower in Tg338 mice than in the natural animal model, explaining only 13.69% of variation (*p* < 0.029), whereas interindividual variation constituted 15.57% of variation (*p* < 0.0001), being the highest effect for the area analysed (48.37% variation, *p* < 0.0001). After Bonferroni correction for multiple tests, a significant difference of BAMBI staining was observed in DCN (*p* < 0.05) between controls and scrapie-infected mice at the clinical stage of the disease. Effect of disease was even smaller when clinical and preclinical scrapie groups were compared (7.22% of variation, *p* < 0.001), even though this outcome should be taken with caution as the ANOVA test was performed with a limited number of subjects (*n* = 3 in each group). After Bonferroni correction, a significant difference was observed in Tc (*p* < 0.05). 

**Figure 6:**
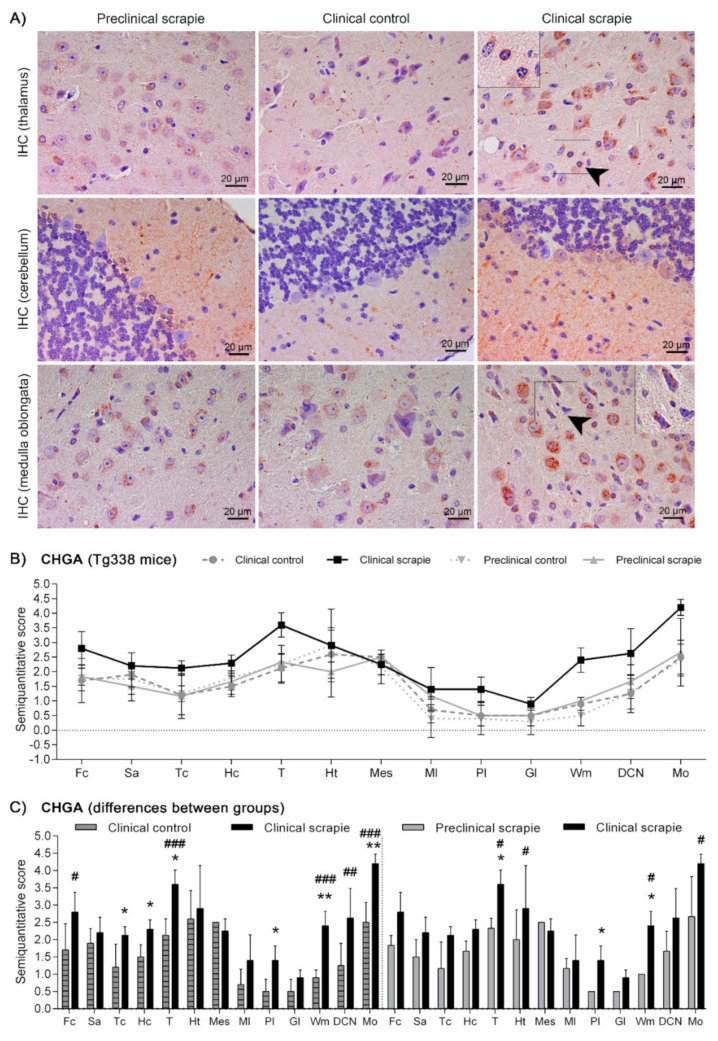
Immunostaining patterns and semi-quantitative scoring of CHGA in the central nervous system of scrapie-infected Tg338 mice. (**A**) Representative images of CHGA immunostaining in the thalamus, cerebellum and medulla oblongata (20 µm). This protein displayed a linear punctiform synaptic-like immunostaining in the neuropil throughout the brain. In addition, multiple cells exhibited intracytoplasmic granular immunolabelling, being particularly well-defined and strong in the thalamus and medulla oblongata. In the cerebellum, clinically-affected mice showed a mild intracytoplasmic staining of the Purkinje cells, which were not immunolabelled in the rest of the groups. However, these cells occasionally had partially stained neurites, not only in clinical mice, but also in the preclinical and control group. Notice the glial cell immunolabelling (arrowheads and detail). Figure includes a global graph (**B**) and comparative graphs (**C**) between clinical group (black bars) with preclinical (grey bars) and control group (grey-striped bars). Comparative graphs between the preclinical group and their control group, and between control groups, are not shown because no significant differences were detected. Score values (from 0: negative, to 5: staining present at its maximum intensity) evaluated in the frontal cortex (Fc), septal area (Sa), thalamic cortex (Tc), hippocampus (Hc), thalamus (T), hypothalamus (Ht), mesencephalon (Mes), cerebellum (which includes molecular layer (Ml), Purkinje layer (Pl), granular layer (Gl), white matter (Wm) and deep cerebellar nuclei (DCN)) and medulla oblongata (Mo). The differences between the experimental groups were determined using the Mann Whitney U test (*p < 0.05 and **p < 0.01). Significant differences after Bonferroni correction for multiple tests are shown as # *p* < 0.05, ## *p* < 0.01 and ### *p* < 0.001.

Protein CHGA in Tg338 mice presented an immunostaining pattern similar to that described for scrapie sheep (Figure 6A). A linear punctiform synaptic-like immunostaining was observed in the neuropil throughout the brain. In addition, multiple cells exhibited intracytoplasmic granular immunolabelling, being particularly well-defined and strong in certain regions of clinical mice, such as T, DCN and Mo. The morphology of some CHGA-immunopositive cells was compatible with glial cells. In Cbl, clinically-affected mice showed a mild intracytoplasmic staining of the Purkinje cells, which were not immunolabelled in the other groups. However, these cells occasionally had partially stained neurites, not only in clinical mice, but also in the preclinical and control group. Like BAMBI, CHGA deposition scores were higher in clinically-infected mice (Figure 6B). This increase was statistically significant in Tc (*p* = 0.039), Hc (*p* = 0.023), T (*p* = 0.015), Pl (*p* = 0.023), Wm (*p* = 0.007) and Mo (*p* = 0.007) (Figure 6C). The effect of disease explained 20.38% of the variation between scrapie-infected mice at the clinical stage and their controls (*p* < 0.0006), and after Bonferroni correction, changes were still significant at Fc (*p* < 0.05), T (*p* < 0.001), Wm (*p* < 0.001), DCN (*p* < 0.01) and Mo (*p* < 0.001). Moreover, when clinical animals were compared to the preclinical-stage group, a significant increase of CHGA was observed in T (*p* = 0.017), Pl (*p* = 0.035) and Wm (*p* = 0.035). The two-way ANOVA confirmed that the effect of disease corresponds to 20.50% of total variation. Statistically significant differences between areas after correction for multiple tests are shown in Figure 6C.

### 3.3. Western Blot Quantification of BAMBI and CHGA Proteins in the Central Nervous System of Scrapie-Affected Sheep

**Figure 7:**
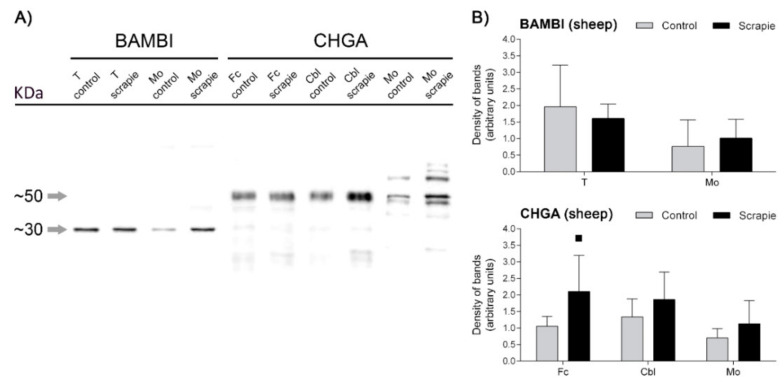
Western blot quantification of BAMBI and CHGA proteins in the CNS of scrapie-infected sheep. (**A**) Membranes displaying the specificity of antibodies against BAMBI and CHGA proteins in the ovine thalamus (T), medulla oblongata (Mo), frontal cortex (Fc) and cerebellum (Cbl) of control and scrapie-infected sheep detected using Western blot. Each protein was cropped and grouped from different parts of the same gel or from different gels. Distinctive bands of ~30 kDa and ~50 kDa confirmed the specificity of the antibodies used against BAMBI and CHGA, respectively. (**B**) Western blot quantification of BAMBI in T and Mo and CHGA in Fc, Cbl and Mo. Density of immunoreactive bands was normalized for β-Actin density band and is reported as arbitrary units. Data are expressed as means ± standard deviation. Quantification of density of bands did not reveal significant changes in any of the analysed areas, although CHGA displayed a trend to upregulation in scrapie animals (black bars) in Fc (*p* = 0.097). Differences between groups were determined using the Student´s *t*-test (▪ *p* < 0.1).

We performed a protein quantification of BAMBI and CHGA using Western blot in those brain areas of sheep that displayed significant immunohistochemical changes between groups. A unique and specific band at ~30 kDa was detected for BAMBI, whereas CHGA revealed a multiband pattern with a major band migrating at a molecular mass of ~50 kDa (Figure 7A). Quantification of the density of the bands did not reveal significant changes in any of the analysed areas, although CHGA displayed a trend to upregulation in scrapie animals in Fc (*p* = 0.097) (Figure 7B).

### 3.4. BAMBI and CHGA Expression Positively Correlates with Prion Neuropathology in Scrapie

As described above, BAMBI and CHGA distribution was not identical but the scores of these proteins were always higher in scrapie animals. In fact, we observed a strong correlation between BAMBI and CHGA levels in the total set of animals and within individual groups (Table 1), suggesting a coordinated regulation of these proteins in vivo. To analyse the relationship between these proteins and prion neuropathology in ovine scrapie, we compared their immunohistochemical scores with previously described neuropil spongiosis, intraneuronal vacuolation and PrP^Sc^ deposition patterns. Briefly, histopathological analysis revealed a significant increment of neuropil spongiosis and intraneuronal vacuolation in most brain areas of scrapie sheep, and the misfolded PrP^Sc^ protein was only detected in these animals [17]. Activated microglial cells analysed in this study displayed a significant increase in basal ganglia (Bg), T, pons (P) and Mo, showing the highest scores for the three latter aforementioned areas (Figure S2A). The scores of BAMBI and CHGA immunostaining positively correlated with all scrapie-related lesions (Table 1), the strongest correlation for the levels of both proteins being with PrP^Sc^ deposition in the total set of sheep (ρ = 0.409 for BAMBI and ρ = 0.507 for CHGA, *p* < 0.001) and with spongiosis in the scrapie group (ρ = 0.563 for BAMBI and ρ = 0.564 for CHGA, *p* < 0.001). 

Histopathological profiles in clinical Tg338 mice were compatible with TSE and resembled those observed in classical scrapie sheep. All mice sacrificed due to clinical disease were positive for PrP^Sc^ in the brain and showed the highest accumulation of spongiform changes in T, Ht, Mes and Mo [20]. Microgliosis scores were very similar between the four groups analysed, although Hc, T, Ht and Mo displayed a slight but significant staining increment in clinical mice (Figure S2B). As mentioned before for scrapie sheep, the scores of BAMBI and CHGA immunostaining in Tg338 mice positively correlated with scrapie-related lesions (Table 1). In clinical mice, correlations were stronger with spongiosis and microgliosis (ρ > 0.5, *p* < 0.001) than with prion deposition (ρ < 0.5, *p* < 0.01). In addition, the two proteins positively correlated with each other in all groups analysed.

### 3.5. BAMBI Is Upregulated in CSF of Scrapie-Affected Sheep at Clinical Stage and CJD Patients

**Figure 8:**
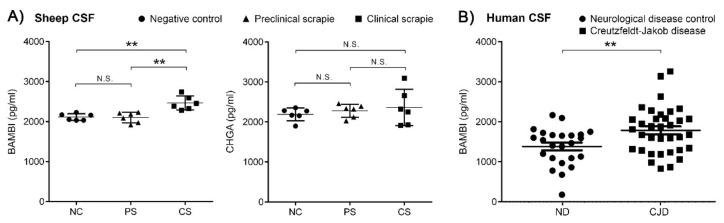
ELISA quantification of BAMBI and CHGA proteins in CSF of scrapie-infected sheep and BAMBI in CSF of CJD patients. (**A**) Intensity of BAMBI and CHGA signals in CSF of control (NC, round dots) and scrapie sheep at preclinical (PS, triangular dots) and clinical stage (CS, square dots). Protein BAMBI displayed an upregulation in CSF of scrapie-affected sheep at clinical stage (2465 ± 174 pg/mL) compared to scrapie sheep at preclinical stage (2103 ± 132 pg/mL, *p* < 0.01) and to control sheep (2114 ± 84 pg/mL, *p* < 0.01). No significant differences were detected between groups for CHGA protein. (**B**) Concentrations of BAMBI in CSF of control (ND, round dots) and CJD cases (square dots). This protein displayed increased concentrations in CJD (1771 ± 588 pg/mL) compared to ND (1370 ± 474 pg/mL, *p* < 0.01). N.S.: no statistically significant value. Differences between groups were determined using the Student´s *t*-test (** *p* < 0.01).

In order to test their potential as biomarkers, the intensity of BAMBI and CHGA signals were quantified using ELISA in the CSF of control and scrapie sheep at the clinical and preclinical stages of the disease. Protein BAMBI displayed an upregulation in the CSF of scrapie sheep at the clinical stage (2465 ± 174 pg/mL) compared to those at the preclinical stage (2103 ± 132 pg/mL, *p* < 0.01) and to control sheep (2114 ± 84 pg/mL, *p* < 0.01) (Figure 8A and Table S2). No significant differences were detected between groups for CHGA protein.

We selected BAMBI for further analysis in CSF from CJD patients. Quantification of BAMBI revealed increased concentrations in CJD (1771 ± 588 pg/mL) compared to ND (1370 ± 474 pg/mL, *p* < 0.01) (Figure 8B and Table S2). Cases of CJD presented a typical CSF prion biomarker profile with increased t-tau concentrations and 14-3-3 positivity compared to controls (Table S2). Concentrations of BAMBI in CSF were not statistically different between CJD cases harbouring methionine/methionine [MM] (1750 ± 632 pg/mL, *n* = 11), methionine/valine [MV] (1833 ± 506 pg/mL, *n* = 7) and valine/valine [VV] (1672 ± 624 pg/mL, *n* = 11) at codon 129 of the *PRNP* gene (*p* > 0.05). Additionally, no association between t-tau and BAMBI were detected in CJD cases (r = 0.03, *p* > 0.5), suggesting that CSF BAMBI is not a surrogate marker of neuro-axonal damage.

## 4. Discussion

The complexity of neuropathology associated to prion diseases requires an interdisciplinary approach for its understanding. A genomic study performed in vitro using cell lines with different susceptibilities to prion replication determined potential genes involved in the susceptibility to PrP^Sc^ propagation [9]. Some genes involved in ECM homeostasis were expressed by prion-resistant revertants of highly susceptible neuroblastoma cells, and their expression was downregulated after treatment with retinoic acid, which was linked to an augmented prion replication [9]. The aim of our study was to investigate the implication of a battery of those genes in the neuropathology associated with prion diseases in vivo. As animal models we used sheep naturally infected with classical scrapie and transgenic Tg338 mice that overexpress the highly susceptible variant of the ovine *PRNP* gene [19]. The murine model allows the study of the preclinical stages of the disease, and the neuropathology of infected mice resembles the natural disease [20], however the natural model could better mimic the heterogeneity of human diseases.

We first analysed the expression of these genes in Mo of sheep naturally infected with classical scrapie, since this tissue is one of the most affected by both prion accumulation and spongiform degeneration in this disorder [17,21]. Although most of the genes trend to reduce their expression in scrapie medullae, statistically significant downregulation was only observed for *CHGA*. The observed lack of significance can be by some means expected due to the cellular complexity of the CNS, where all cellular types are not necessarily involved in prion propagation. Moreover, the *Chga* gene was also downregulated in the Mes of Tg338 mice at the clinical stage, but not at the preclinical stage, suggesting that the regulation of this gene occurs late in prion neuropathology. 

Whilst we found the CHGA (chromogranin A) protein mainly forming coarse intracytoplasmic aggregates within the neurons of natural scrapie and scrapie-infected transgenic mice, this protein locates at the ECM level in prion-resistant cell cultures [9]. Although the role of CHGA as a modulator of cell adhesion has been suggested [9,30], its immunohistochemical determination is in accordance with its known function as a neuroendocrine secretory glycophosphoprotein. This protein belongs to a multifunctional protein family widely enclosed within large dense-core secretory vesicles in the neuroendocrine system and a variety of neurons, where they constitute the main protein component of the intravesicular matrix [31]. 

Changes in transcript levels were not reflected at protein level. In contrast to the in vitro model, where downregulation of *CHGA* transcription and translation was associated with a significant increase in prion propagation [9], we found a significant overexpression of this protein in some CNS areas of scrapie-infected animals at the clinical stage for both the natural and the transgenic model. This protein is processed into derived peptides in the ECM, which exert regulatory activities [11]. In our work, immunostaining was more intense in the ECM of scrapie animals, suggesting that derivative peptides could play a role in this neurodegenerative disease. In addition, the scores of the CHGA immunostaining positively correlated with prion-related lesions in both scrapie models. Similarly, the levels of *CHGA* are elevated in the temporal cortex from AD patients [32,33], and the presence of a large amount of this protein around β-amyloid plaques suggests that neurosecretory granules could be involved in β-amyloid formation [34]. 

In vitro experiments support that CHGA is one of the endogenous factors that trigger the microglial response involved in neuronal degeneration, thus being an important stimulator of neuroinflammation [35,36]. It has also been postulated as a potent pro-inflammatory inducer of neuroinflammation in AD [37,38], since it activates microglial stress pathways [34]. In scrapie-infected animals CHGA accumulated in some brain areas associated with reactive microglia, i.e., Mo in sheep, and Hc, T and Mo in Tg338 mice. Moreover, in clinical Tg338 mice we detected a stronger correlation between the CHGA immunostaining scores and microgliosis than that observed with prion deposition, which suggests that this protein is likely to be a mediator between inflammatory and neurodegenerative mechanisms also in prion diseases.

In addition to AD [34], CHGA also accumulates in brain areas with neuronal degeneration in Parkinson´s [39] and Pick´s disease [40], suggesting that this protein could be a useful biomarker for various neurological disorders. In fact, the potential of CHGA as biomarker in CSF has been investigated in prion-like neurodegenerative diseases such as AD [34] or amyotrophic lateral sclerosis [41]. Although we did not find statistical differences of CHGA concentrations in the CSF of scrapie sheep and its upregulation in other neurodegenerative diseases diminishes its potentiality as a prion biomarker, data in the CNS of both scrapie models indicate that expression of this protein is a key feature also in prion diseases.

The other gene dysregulated in scrapie was *BAMBI*. As previously shown in vitro [9], *Bambi* was downregulated in Mes from clinically-affected Tg338 mice. However, this gene was overexpressed in Mo of scrapie-infected sheep. As the expression analysis was performed in different tissues, the difference between these two models could be related to a difference in lesion degree or to the temporality of events associated with the pathology. Nevertheless, the encoded protein was significantly overexpressed in the CNS of natural scrapie animals and clinically-infected mice. In both models, BAMBI positively correlated with scrapie-related lesions. We observed an intranuclear immunostaining of BAMBI in neurons and glial cells in all brain areas. Immunostaining of BAMBI in both cytoplasm and nucleus has been previously described in other organs such as porcine ovary [42]. In fact, co-translocation of BAMBI with SMAD proteins, transcriptional co-regulators involved in TGF-β signalling, into the nucleus in tumoral ovarian cells has been associated with a modulation of TGF-β signalling in the nucleus rather than with an inhibition [43]. The immunostaining observed in our work might be related to the regulation of this pathway. In addition, immunostaining for this protein in neuropil was also stronger in scrapie animals. Although this protein modulates ECM deposition in some tissues through TGF-β regulation [44], the presence of this protein in ECM has not been described before. Further studies are necessary to elucidate whether the extracellular fraction of this transmembrane protein could interact with ECM proteins, which could explain this staining.

The TGF-β overexpression occurs in the CNS of patients with sporadic CJD, although changes differ between subtypes of this disease [45]. In natural ovine scrapie, a gene expression analysis of TGF-β did not reveal significant changes in the CNS [46], although genes involved in the TGF-β signalling pathway are dysregulated in bovine spongiform encephalopathy medullae [47]. More interesting, it has been suggested that the impairment of TGF-β signalling and inhibition of its anti-inflammatory properties could prolong microglial activation and contribute to the development and maintenance of neuroinflammation [48]. Although β-amyloid (1-42) oligomers suppress *TGF-β* gene expression in vitro inducing a transient upregulation of *Bambi* [48], the role of BAMBI protein in other neurodegenerative diseases has not been further investigated. The upregulation observed in our study in scrapie medullae at the transcript level, the increment of BAMBI immunostaining in several CNS areas of both scrapie models and its positive correlation with scrapie-related lesions suggest a role of this protein in prion neuropathology. Overexpression of this molecule could be involved in the regulation of chronic neuroinflammation in scrapie, possibly through the regulation of TGF-β expression. 

Finally, although both analysed proteins, BAMBI and CHGA, were overexpressed in the CNS of scrapie-infected animals, only BAMBI levels were significantly upregulated in the CSF of scrapie sheep at the clinical stage. In order to evaluate the possible translation of our findings to human medicine, BAMBI was quantified in CSF from CJD patients and found increased. Although the overlap between the CJD and control groups was high and, thus, limits its use as a diagnostic biomarker, this analysis confirmed the potential role of BAMBI in human prionopathies and the utility of the natural ovine model for the study of prion neuropathology and the identification of potential biomarkers. Further research is still required to elucidate the molecular pathways in which BAMBI and CHGA could be participating in prion disease-related conditions.

## 5. Conclusions

Using natural ovine scrapie, our study certainly suggests that BAMBI and CHGA could play a relevant role in TSE neuropathology in vivo and may be involved in the characteristic neuroinflammatory response associated to these disorders. Moreover, our work also supports the potential use of BAMBI as a biomarker to detect clinical scrapie and human prion disease cases. This research contributes to a more profound understanding of the molecular pathways involved in neuroinflammation and reveals potential targets for improving chronic inflammation, which could help to develop therapeutic interventions for neurodegenerative diseases.

## Figures and Tables

**Table 1:** Spearman correlation values between BAMBI and CHGA immunostaining scores and scrapie-related histopathological lesions (spongiosis, intraneuronal vacuolation, PrP^Sc^ deposition and microgliosis) in sheep (in the total set of sheep and only in scrapie-infected sheep) and Tg338 mice (in the preclinical and clinical stage, in the total set of animals and only in scrapie-inoculated mice). Correlations were estimated using the full set of data obtained in all tissues.

	Sheep		Tg338 Mice					
			Preclinical Stage		Clinical Stage		Total Set	Scrapie Clinical + Preclinical
	Control + Scrapie	Scrapie	Control + Scrapie	Scrapie	Control + Scrapie	Scrapie		
BAMBI								
Spongiosis	0.362 ***	0.563 ***	0.379 ***	0.545 ***	0.559 ***	0.709 ***	0.483 ***	0.648 ***
Intraneuronal vacuolation	0.392 ***	0.447 ***	----	----	----	----	----	----
PrP^Sc^ deposition	0.409 ***	0.540 ***	----	N.S.	----	0.454 **	----	0.327 *
Microgliosis	0.313 ***	0.489 ***	0.487 ***	0.515 **	0.371 ***	0.535 ***	0.422 ***	0.575 ***
CHGA								
Spongiosis	0.431 ***	0.564 ***	0.518 ***	0.625 ***	0.668 ***	0.694 ***	0.594 ***	0.694 ***
Intraneuronal vacuolation	0.345 ***	0.476 ***	----	----	----	----	----	----
PrP^Sc^ deposition	0.507 ***	0.530 ***	----	N.S.	----	0.454 **	----	0.550 ***
Microgliosis	0.360 ***	0.451 ***	0.559 ***	0.543 ***	0.724 ***	0.609 ***	0.644 ***	0.662 ***
BAMBI/CHGA	0.702 ***	0.765 ***	0.603 ***	0.637 ***	0.520 ***	0.613 ***	0.545 ***	0.645 ***

N.S.: no statistically significant value. Spearman correlation: * *p* < 0.05, ** *p* < 0.01 and *** author *p* < 0.001.

## Supplementary Materials

The following are available online at https://www.mdpi.com/2218-273X/10/5/706/s1, Figure S1: Scrapie-associated histopathology in medulla oblongata of sheep, Figure S2: Microgliosis in the CNS of scrapie-affected sheep and scrapie-infected Tg338 mice, Table S1: Sheep used for the gene expression study, immunohistochemical assays and ELISA analysis, Table S2: Demographic and biomarkers data from the study cohort, Table S3: Primers used for the amplification of candidate genes in sheep, Table S4: Correlation values between scrapie-related lesions in medulla oblongata of scrapie-affected sheep and expression of genes potentially involved in prion replication.
