# Supplementary material for: BAMBI and CHGA in Prion Diseases: Neuropathological Assessment and Potential Role as Disease Biomarkers
Source: Biomolecules. 2020 May 2;10(5):706. doi: 10.3390/biom10050706 (PMC7277700; doi:10.3390/biom10050706)
Supplement: Supplementary file 1 [file biomolecules-10-00706-s001.pdf]

Table S1. Sheep used for the gene expression study, immunohistochemical assays and ELISA analysis. Characteristics of these animals (type of assay, clinical stage, number of cases, tissue employed, breed, sex, age and genotype), as well as the reference of the works in which they were previously used are indicated.

Assay	Clinical stage	n	Tissue	Breed	Sex (f/m)	Age (years)	Genotype	Reference
**qPCR**	NC	5	Mo	Rasa Aragonesa	5/0	7.51 ± 1	ARQ/ARQ	----
	CS	5			5/0	3.39 ± 1.26		[18]
**IHQ**	NC	6	CNS	Rasa Aragonesa	6/0	4.06 ± 1.41	ARQ/ARQ	[17]
	CS	6			6/0	4.69 ± 0.48		
**ELISA**	NC	6	CSF	Rasa Aragonesa	6/0	5.66 ± 0.94	ARQ/ARQ	----
	PS	6			6/0	3.17 ± 1.34		----
	CS	6			6/0	5.17 ± 0.68		----

n: number of cases; f: female; m: male; IHQ: immunohistochemistry; NC: negative control; CS: clinical scrapie; PS: preclinical scrapie; Mo: medulla oblongata; CNS: central nervous system; CSF: cerebrospinal fluid.

Table S2. Demographic and biomarkers data from the study cohort. BAMBI and CHGA concentrations (in pg/ml) as well as t-tau concentration (in pg/ml) and 14-3-3 positivity (positive/negative) are indicated.

Sample	Clinical stage	n	Sex (f/m)	Age (years)	BAMBI (Mean±SD, pg/ml)	CHGA (Mean±SD, pg/ml)	t-tau (Mean±SD, pg/ml)	14-3-3 (positive/negative)
**Sheep CSF**	NC	6	6/0	5.66 ± 0.94	2114 ± 0.08	2189 ± 0.06	----	----
	PS	6	6/0	3.17 ± 1.34	2103 ± 0.13	2278 ± 0.06	----	----
	CS	6	6/0	5.17 ± 0.68	2465 ± 0.17	2362 ± 0.18	----	----
**Human CSF**	ND	24	11/13	67 ± 8	1370 ± 474	----	368 ± 355	4/20
	CJD	34	17/17	65 ± 5	1771 ± 588	----	8765 ± 8707	31/3

t-tau: total tau; n: number of cases; f: female; m: male; SD: standard deviation; CSF: cerebrospinal fluid; NC: negative control; PS: preclinical scrapie; CS: clinical scrapie; ND: neurological disease control; CJD: Creutzfeldt-Jakob disease.

Table S3. Primers used for the amplification of candidate genes in sheep. Ensemble access numbers for the ovine genes (ENSOARG) are shown in brackets under their corresponding gene name.

Gene (ENSOARG)	Primer sequences	Primer length (bp)	Tm (°C)	Amplicon length (bp)
IL11RA	Fw: 5′ GTTGCCCTGGAGTGACTGTT 3′	20	60.2	88
(00000009133)	Rv: 5′ CCTAGCCCAGAGGCAGGT 3′	18	60.4	
RGS4	Fw: 5′ ATCTCAGTCCAGGCAACCAA 3′	20	60.7	103
(00000011014)	Rv: 5′ GAGCCTCATCGAAGCAAGTT 3′	20	59.6	
PAPSS2	Fw: 5′ AGAGGTGGCCAAGCTGTTT 3′	19	59.9	107
(00000014018)	Rv: 5′ CTGCCGATTCATGGATTTTG 3′	20	61.4	
BAMBI	Fw: 5′ GGCTACAGGATGTCCTCACC 3′	20	59.5	118
(00000015180)	Rv: 5′ TCTTTGGAGGACGTCAGCTC 3′	20	60.5	
DLC1	Fw: 5′ TCTATGGCGATTCCAGGTCT 3′	20	59.7	98
(00000009748)	Rv: 5′ GCATACTGGGGGAATCCTG 3′	19	59.7	
LRRN4	Fw: 5′ CTACGGGTCCTTCATTTCCA 3′	20	59.9	92
(00000018440)	Rv: 5′ CCGAAGAGGTTGATGGACAG 3′	20	60.7	
ITGA8	Fw: 5′ TGCTGTGGCACATTTAGGAG 3′	20	59.9	98
(00000008389)	Rv: 5′ TGAGCACTTTGCCTCTTTGA 3′	20	59.7	
CHGA	Fw: 5´ TGTATCGTCGAGGTCATCTCTGA 3´	23	58.4	144
(00000013347)	Rv: 5´ CGAGGTCTTGGAGCTCTTTCA 3´	21	58.3	
FN1	Fw: 5´ GGCTGAACCGGGTAACGAA 3´	19	59.5	111
(00000019329)	Rv: 5´ AGGCATGAAGCACTCAATTGG 3´	21	58.8	
GALT	Fw: 5´ ACCCCCACAACCCTCTCTGT 3´	20	59.3	100
(00000009164)	Rv: 5´ CAGAGCTGGGAAGTCGTTGTC 3´	21	58.5	

bp: base pair; Tm: melting temperature; Fw: forward; Rv: reverse.

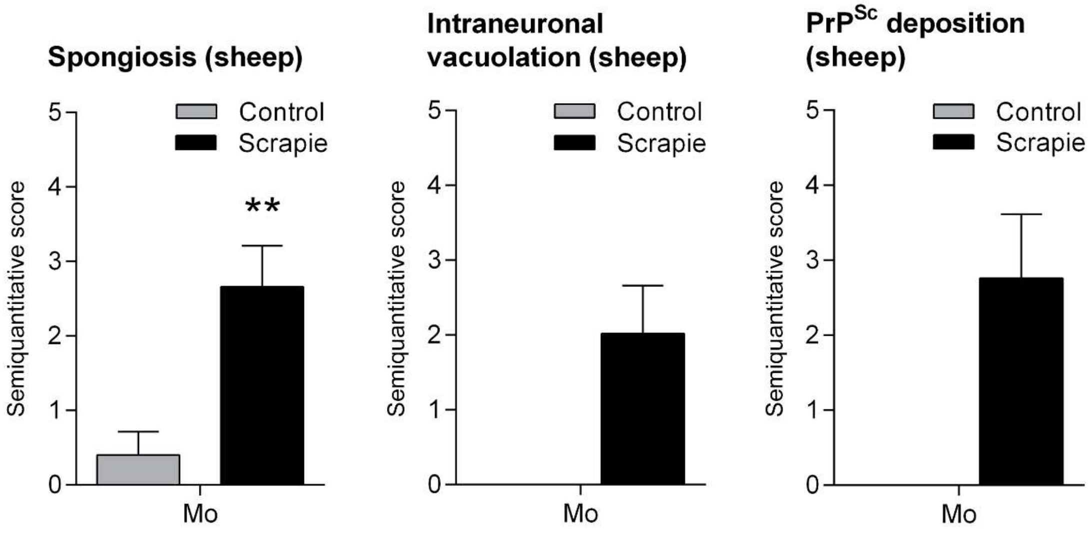

Figure S1. Scrapie-associated histopathology in medulla oblongata of sheep. Graphics show the semi-quantitative assessment values of spongiosis, intraneuronal vacuolation and PrP^{Sc} deposition in medulla oblongata (Mo) of scrapie (black bars) and control sheep (grey bars). Scores range from 0 (negative) to 5 (lesion or staining present at maximum intensity). Significant differences were determined using the Mann Whitney U test (**$P < 0.01$).

Table S4. Correlation values between scrapie-related lesions in medulla oblongata of scrapie-affected sheep and expression of genes potentially involved in prion replication. Spearman (ρ) correlation was calculated in the total set of sheep and Pearson (r) correlation was calculated only in scrapie animals.

	**PrP^{Sc} deposition**		**Intraneuronal vacuolation**		**Spongiosis**	
**Gene**	**Total set (ρ)**	**Scrapie (r)**	**Total set (ρ)**	**Scrapie (r)**	**Total set (ρ)**	**Scrapie (r)**
BAMBI	**0.83 (P = 0.003)**	**0.98 (P = 0.003)**	**0.692 (P = 0.027)**	-0.205 (N.S.)	**0.705 (P = 0.023)**	-0.181 (N.S.)
CHGA	**-0.892 (P = 0.001)**	-0.415 (N.S.)	**-0.836 (P = 0.005)**	-0.01 (N.S.)	**-0.733 (P = 0.025)**	0.853 (P = 0.066)
DLC1	0.586 (N.S.)	0.565 (N.S.)	0.592 (N.S.)	0.444 (N.S.)	0.426 (N.S.)	0.065 (N.S.)
FN1	-0.195 (N.S.)	-0.813 (P=0.09)	0.019 (N.S.)	0.526 (N.S.)	0.213 (N.S.)	0.666 (N.S.)
GALT	-0.175 (N.S.)	-0.282 (P=0.079)	-0.019 (N.S.)	0.365 (N.S.)	0.177 (N.S.)	**0.962 (P = 0.009)**
IL11RA	-0.506 (N.S.)	-0.724 (N.S.)	-0.136 (N.S.)	0.762 (N.S.)	-0.258 (N.S.)	0.538 (N.S.)
ITGA8	-0.214 (N.S.)	0.569 (N.S.)	-0.304 (N.S.)	0.203 (N.S.)	-0.319 (N.S.)	0.236 (N.S.)
LRRN4	0.367 (N.S.)	0.767 (N.S.)	0.3104 (N.S.)	0.244 (N.S.)	0.317 (N.S.)	-0.047 (N.S.)
PAPSS2	-0.487 (N.S.)	-0.423 (N.S.)	-0.356 (N.S.)	0.427 (N.S.)	-0.307 (N.S.)	**0.968 (P = 0.007)**
RGS4	0.61 (N.S.)	-0.344 (N.S.)	-0.51 (N.S.)	0.308 (N.S.)	-0.384 (N.S.)	**0.938 (P = 0.018)**

N.S.: No statistically significant value.

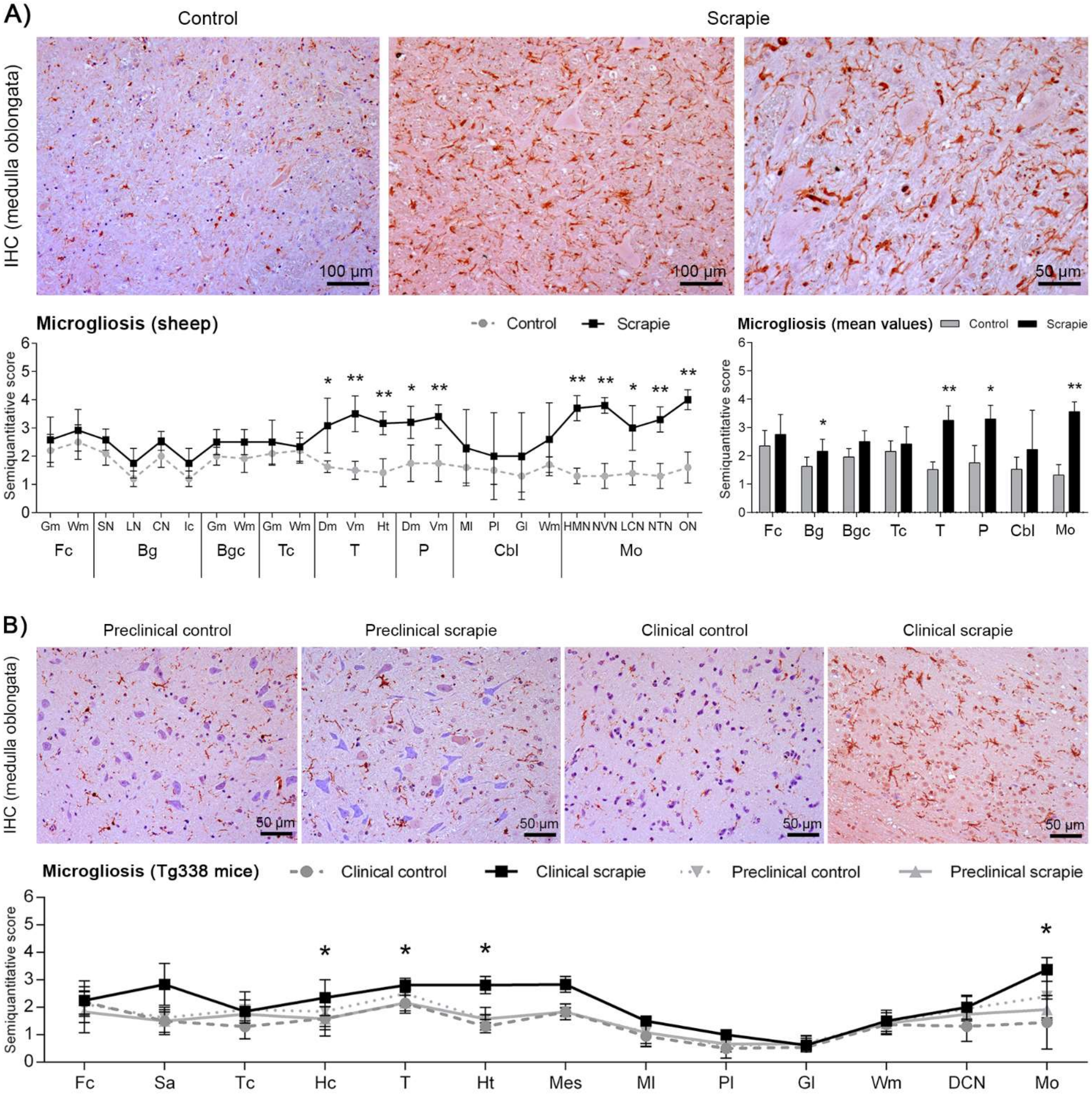

Figure S2. Microgliosis in the CNS of (a) scrapie-affected sheep and (b) scrapie-infected Tg338 mice. Figure shows representative images of reactive microglia in medulla oblongata (100 and 50 μm). Graphics show the semi-quantitative assessment values of microgliosis in different brain areas: frontal cortex (Fc), basal ganglia (Bg), basal ganglia cortex (Bgc), thalamic cortex (Tc), thalamus (T), pons (P), cerebellum (Cbl), medulla oblongata (Mo), grey matter (Gm), white matter (Wm), septal nucleus (SN), lateral nucleus (LN), caudate nucleus (CN), internal capsule (Ic), dorsomedial (Dm), ventromedial (Vm), hypothalamus (Ht), molecular layer (Ml), Purkinje layer (Pl), granular layer (Gl), hypoglossal motor nucleus (HMN), dorsal nucleus of the vagus nerve (NVN), lateral cuneate nucleus (LCN), nucleus of the trigeminal nerve spinal tract (NTN), olivary nucleus (ON), septal area (Sa), hippocampus (Hc), mesencephalon (Mes), deep cerebellar nuclei (DCN). Scores range from 0 (negative) to 5 (staining present at maximum intensity). Significant differences were determined using the Mann Whitney U test (*$P < 0.05$ and **$P < 0.01$).
